# Supplementary material for: Lymphocyte predominant cells detect Moraxella catarrhalis-derived antigens in nodular lymphocyte-predominant Hodgkin lymphoma
Source: Nat Commun. 2020 May 18;11:2465. doi: 10.1038/s41467-020-16375-6 (PMC7235000; doi:10.1038/s41467-020-16375-6)
Supplement: Supplementary file 1 — Supplementary Information [file 41467_2020_16375_MOESM1_ESM.pdf]

Supplementary Information

Lymphocyte predominant cells detect *Moraxella catarrhalis*-derived antigens in nodular lymphocyte-predominant Hodgkin lymphoma

Thurner L. and Hartmann S. et al.,

Supplementary table 1: Primers for amplification and cloning of target antigens (5' to 3')

**Primer**	**Sequence**
rpoC-Start-Sma-s	cccggg atgttgaaagatttattagatatcatgaaaggc
rpoC-AA315-Sma-as	cccggg gccacggcgaccattatc
rpoC-AA300-Sma-s	cccggg atgctacaagaagcagttgatg
rpoC-AA600-Sma-as	cccggg ggtcatctccgtgttacactc
rpoC-AA586-Sma-s	cccggg atgccagaaggcatggcg
rpoC-AA865-Sma-as	cccggg caaagcgcccgatggtac
rpoC-AA851-Sma-s	cccggg atggtcgtgaatgcagatggc
rpoC-AA1145-Sma-as	cccggg atcttttggacggcgtgc
rpoC-AA1130-Sma-s	cccggg atgctgccacgagttgccgac
rpoC-Stop-Sma-as	cccggg ctcttgagaacccagctcatg
MID-HAG AA1-SmaI-s	cccggg atg gtgatcggtgcaacgctcaatggc
MID-HAG AA761-SmaI-as	cccggg gccattggcaaagttgacggtgtc
MID-HAG AA920-SmaI-s	cccggg atg ttaaccacgcctaagctgaccgtg
MID-HAG AA1368-SmaI-as	cccggg gccatcggtgccattgatgggctt
MID-HAG AA1353-SmaI-s	cccggg atg tttgtagtaaccgatgctaatggc
MID-HAG AA2090-SmaI-as	cccggg aaagtgaaaacctgcaccaactgc
succ-Start-EcoRV-s	gatatc atgagcgtattagttggaaaag
succ-Stop-EcoRV-as	gatatc ccaaccagtgacttcttttac

<u>Supplementary Table 2: SYFPEITHI prediction of 15mers derived from the BCR binding epitope of RpoC (amino acids 851-865)</u>

<u>A: HLA-DRB1*0401:</u>

**827-841AA:**	**P** V I **V** G **G** **E** I **V** E R L G D R	Score: 20
**832-846AA:**	**G** E I **V** E **R** **L** G **D** R V L G R V	Score: 20
**840-854AA:**	**D** R V **L** G **R** **V** A **A** K D V V N A	Score: 20
**848-862AA:**	**A** K D **V** V **N** **A** D **G** D V V V P S	Score: 20
**849-863AA:**	**K** D V **V** N **A** **D** G **D** V V V P S G	Score: 20

B: <u>HLA-DRB1*0701:</u>

**832-846AA:**	**G** E I **V** E **R** **L** G **D** R V L G R V	Score: 22
**848-862AA:**	**A** K D **V** V **N** **A** D **G** D V V V P S	Score: 22
**849-863AA:**	**K** D V **V** N **A** **D** G **D** V V V P S G	Score: 22
**860-874AA:**	**V** P S **G** A **L** I D **E** R L V E K L	Score: 20
**827-841AA:**	**P** V I **V** G **G** **E** I **V** E R L G D R	Score: 16
**840-854AA:**	**D** R V **L** G **R** **V** A **A** K D V V N A	Score: 16

Supplementary Table 3: Bacterial strains

Staphylococcus aureus	RZ 7 ATCC 29213
Staphylococcus epidermidis	RZ 23 ATCC 14990
Streptococcus pneumoniae	RZ 17 ATCC 49619
Streptococcus pyogenes	RO 355 ATCC 19615
Klebsiella pneumoniae	RO 342 ATCC 1705
Enterobacter cloacae	RO 348 ATCC 700323
Escherichia coli	RZ 47 ATCC 25922
Pseudomonas aeruginosa	PAO 1
Moraxella catarrhalis	RO 108 ATCC 43617<br>and patient isolate
Moraxella osloensis	patient isolate
Haemophilus influenzae	RZ 15 ATCC 49247
Acinetobacter baumannii	19606, RZ Ata +

Supplementary Figures

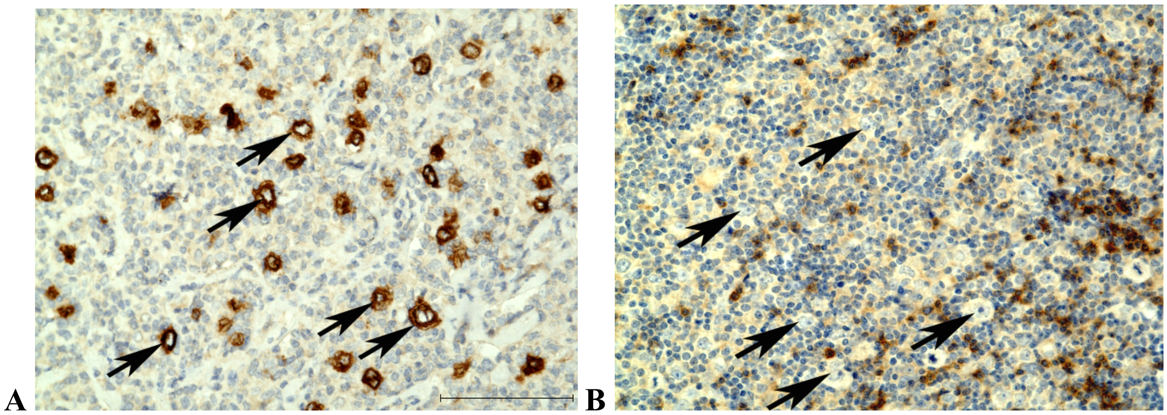

A B

Supplementary Figure 1: Representative immunohistochemical IgD staining of NLPHL. A: IgD^+ NLPHL: LP cells (examples indicated by arrows) show reactivity for IgD (200x). Scale bar size: 100 µm. B: IgD^- NLPHL: LP cells (examples indicated by arrows) show lack of IgD expression whereas few naïve B cells are positive (200x).

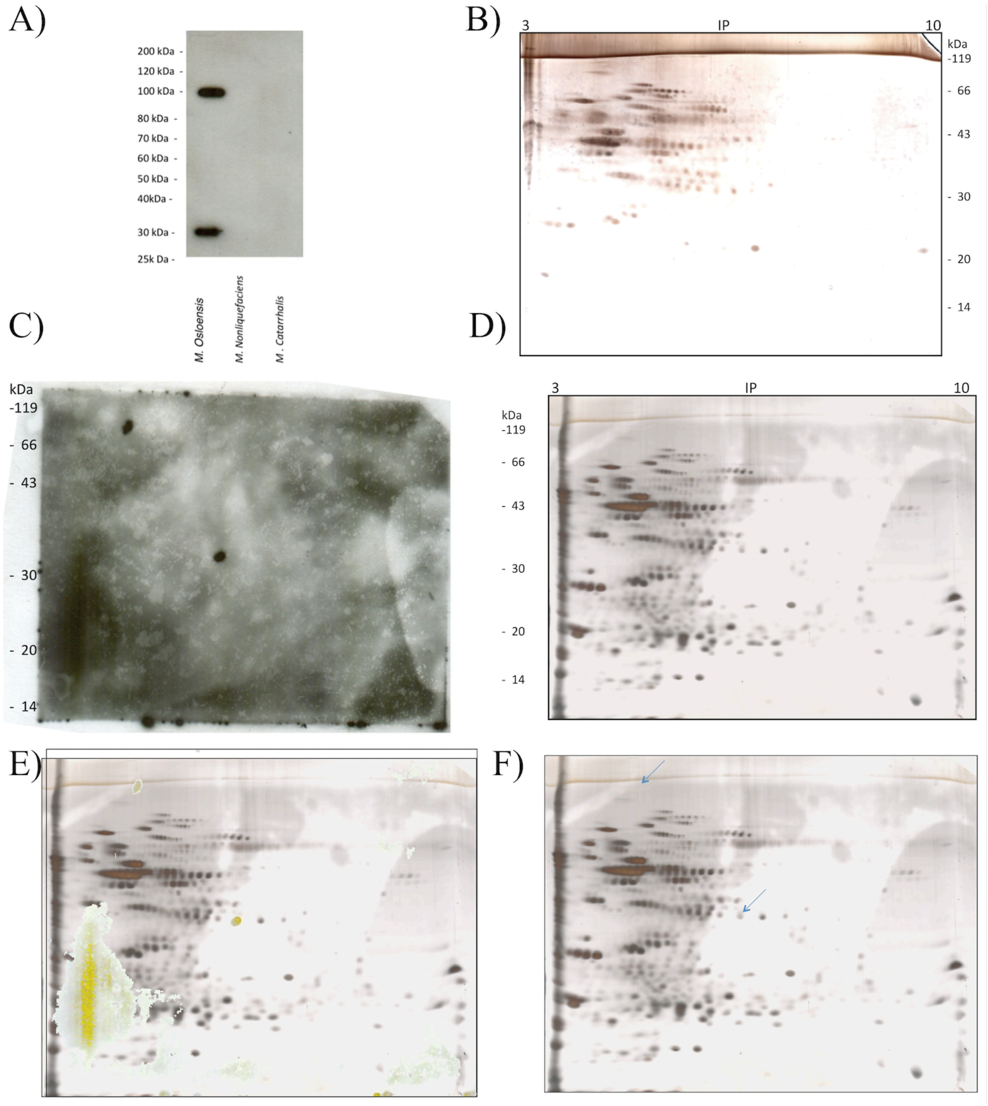

G)

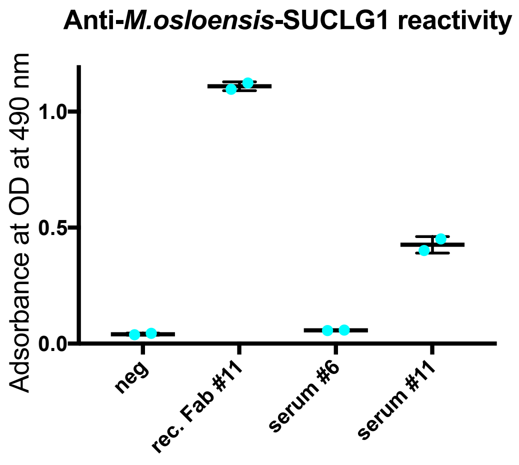

Supplementary Figure 2: Identification of Succinate CoA-ligase subunit α of *M. osloensis* as target antigen of IgD+ NLPHL #11. A: Representative Western blot of simple PAGE of *M.*

osloensis using rec. Fab of #11 as primary antibody showing two antigens. B: Silverstaining of lysate of *M. osloensis* for 45 min of blotting gel (Proteome factory AG, Berlin, Germany). C: Two antigens as dots in 2D gel using the recombinant Fab of patient #11 as primary antibody. D: *M. osloensis* lysate Silverstaining for 5 min of 2D master 2D gel. G: ELISA for reactivity against *M. osloensis* succinate CoA-ligase subunit α. The respective Fab at a concentration of 10µg/ml (positive control = rec. Fab of patient #11) showed a reactivity against succinate CoA-ligase subunit α. The patient was clearly positive for succinate CoA-ligase subunit α antibodies in the serum despite a severe therapy associated hypogammaglobulinemia. Values are mean ± SD of two experiments. Data in A, and G are representative of 2 independent experiments each; data in B to F are derived of a single experiment.

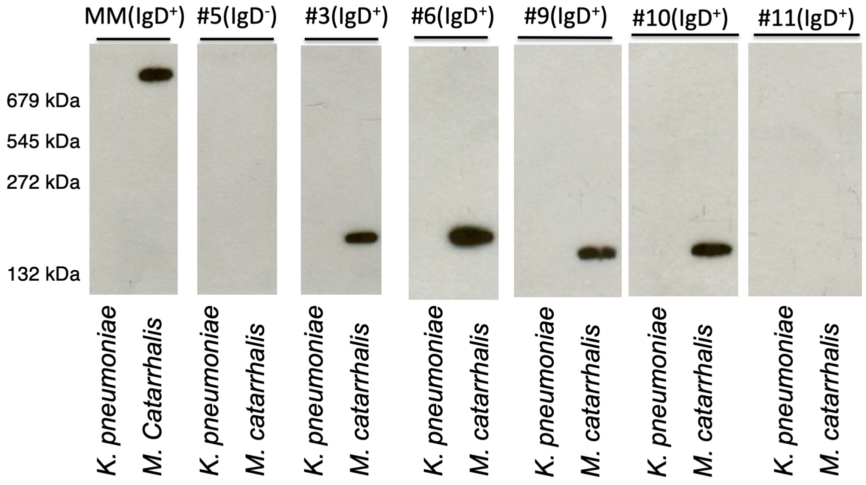

Supplementary Figure 3: Representative Western blots of *M. catarrhalis* lysate under non-reducing conditions. Western blot were developed with individual recombinant NLPHL Fabs or highly diluted serum from a patient with IgD-secreting multiple myeloma (MM) (positive control). A single gel band between 150 – 200 kDa was detected by Fabs #3, #6, #9 and #10. In contrast, serum of the IgD-secreting multiple myeloma detected an 800 kDa band corresponding to the MID/hag tetramer. *Klebsiella pneumoniae* lysate served as negative control. Data are representative of 3 independent experiments.

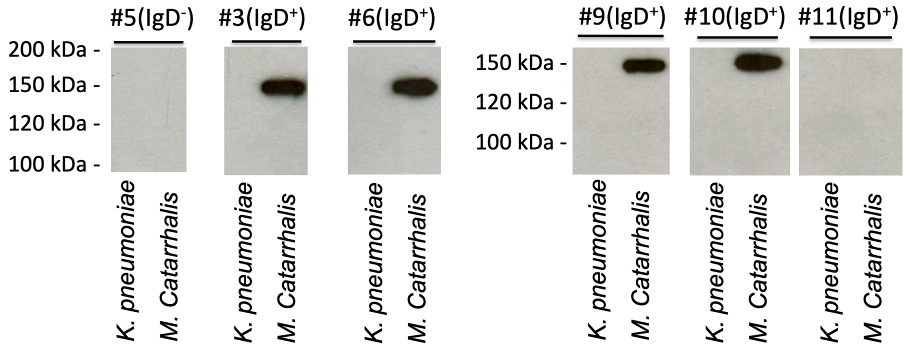

Supplementary Figure 4: Representative Western blot of *M. catarrhalis* lysate under reducing conditions. A specific band between 150 and 200 kDa was detected by individual Fabs of IgD^{+} NLPHL samples #3, #6, #9, and #10. *Klebsiella pneumoniae* lysate served as negative control. Data are representative of 3 independent experiments

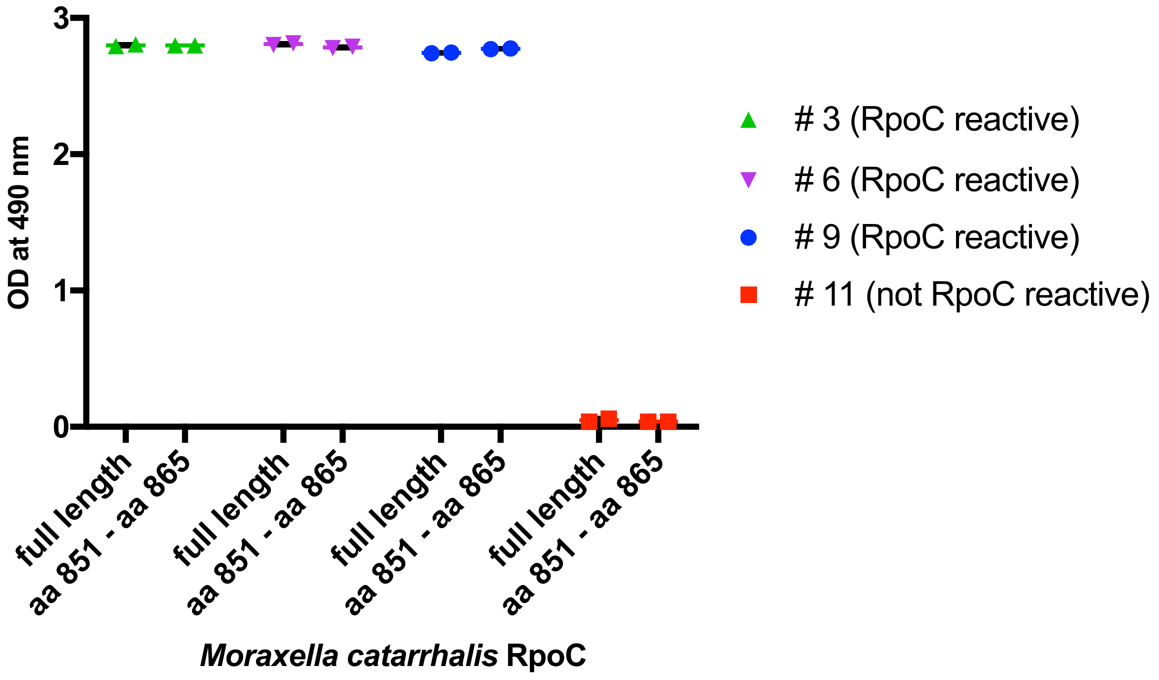

Supplementary Figure 5: Determination of the epitope of RpoC recognized by IgD^{+} NLPHL Fabs. The BCR-binding fragment could be narrowed down to amino acids 851-865 of *M. catarrhalis* RpoC (below). Values are mean ± SD. Data are representative of 2 independent experiments.

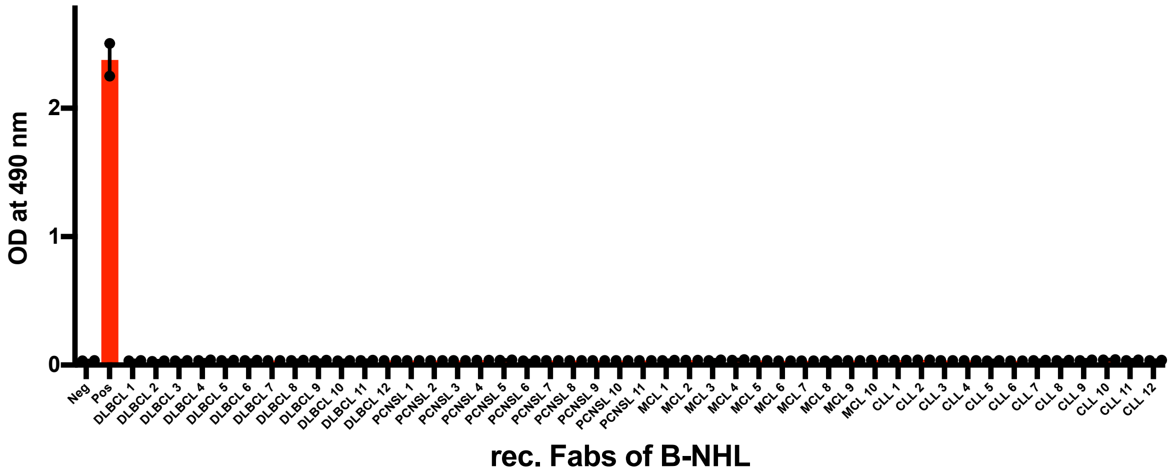

Supplementary Figure 6: Screening of recombinant Fabs of various B-NHLs for reactivity against *M. catarrhalis* RpoC.: Sandwich ELISA for reactivity against *M. catarrhalis* RpoC. Recombinant C-terminally FLAG-tagged RpoC was used as antigen, after initial coating of plates with murine anti-FLAG antibody. Recombinant Fab of NLPHL patient #3 was used as a positive control. Recombinant Fabs of 12 cases of DLBCL, 11 cases of primary central nervous system lymphoma, 10 cases of mantle cell lymphoma and 12 cases of chronic lymphocytic leukemia were screened against *M. catarrhalis* RpoC. Apart from the Fab derived from NLPHL patient #3 no reactivity was detected against *M. catarrhalis* RpoC. Values are mean ± SEM.

A:

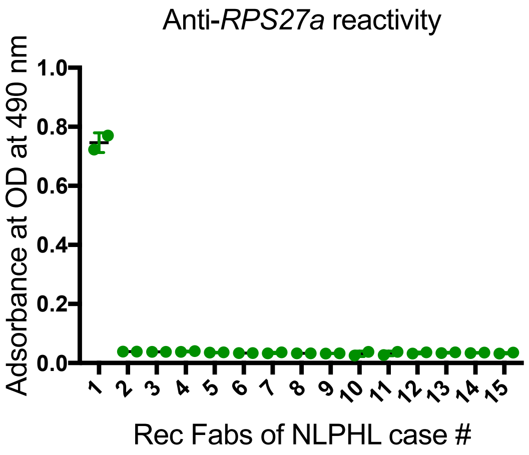

B:

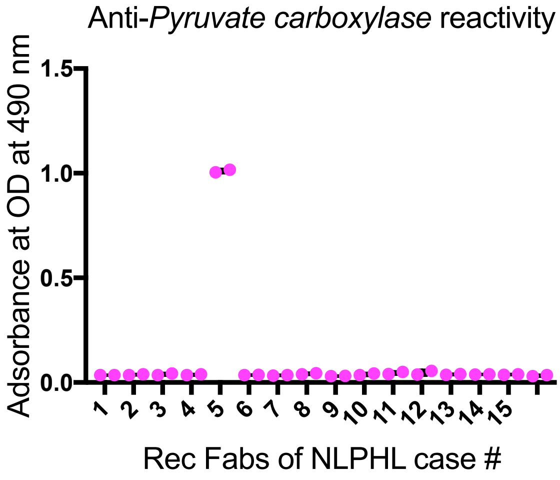
Anti-*Pyruvate carboxylase* reactivity
Adsorbance at OD at 490 nm
1.5
1.0
0.5
0.0
1
2
3
4
5
6
7
8
9
10
11
12
13
14
15
Rec Fabs of NLPHL case #

C:

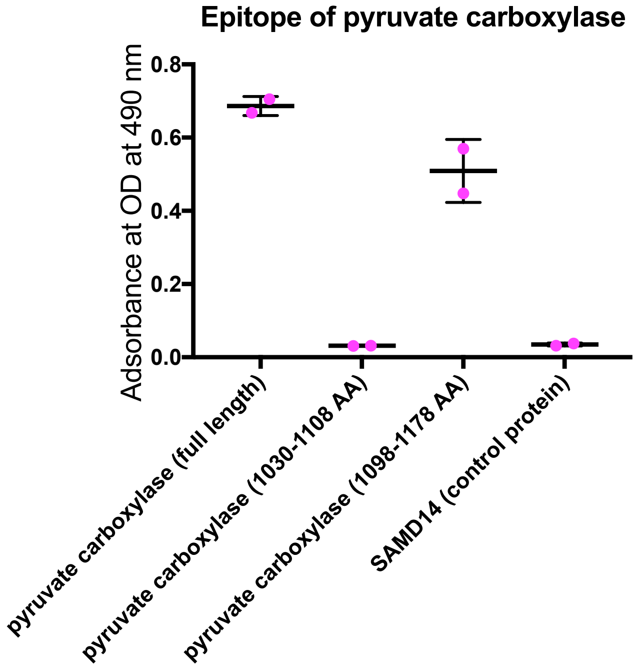
Epitope of pyruvate carboxylase
Adsorbance at OD at 490 nm
0.8
0.6
0.4
0.2
0.0
pyruvate carboxylase (full length)
pyruvate carboxylase (1030-1108 AA)
pyruvate carboxylase (1098-1178 AA)
SAMD14 (control protein)

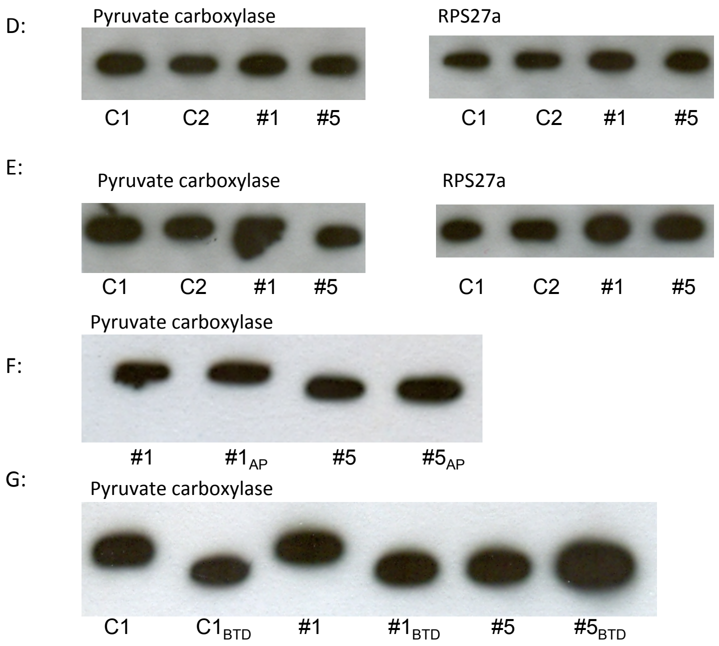

Supplementary Figure 7: BCRs of two IgD^- NLPHL react with autoantigens

Confirmation of candidate autoantigens of lymphoma BCRs by ELISA using recombinant C-terminally FLAG-tagged human RPS27a (A) and human pyruvate carboxylase (B) expressed in HEK293 cells. Values are mean ± SD. Independent experiments were repeated three times. Determination of the epitope region of the pyruvate carboxylase reactive Fab derived from case #5 (C). Values are mean ± SD. Representative Western blots of tissue lysates from two healthy controls and patients #1 and #5 developed with the RPS27a-reactive Fab of patient #1 (D) and the pyruvate carboxylase-reactive Fab of patient #5 as primary antibody showed no difference in molecular weights. Representative IEF demonstrated a less negatively charged pyruvate carboxylase of patient #5 in contrast to patient #1 (E) even after alkaline phosphatase pretreatment (AP) of the tissue lysates (F). Representative IEF after biotinidase pretreatment (BTD) of the tissue lysates showed a disappearance of the different electric charge between the lysate of control 1 or patient #1, with the lysate of patient #5 (G). Data of G-F are representative of 2 independent experiments.

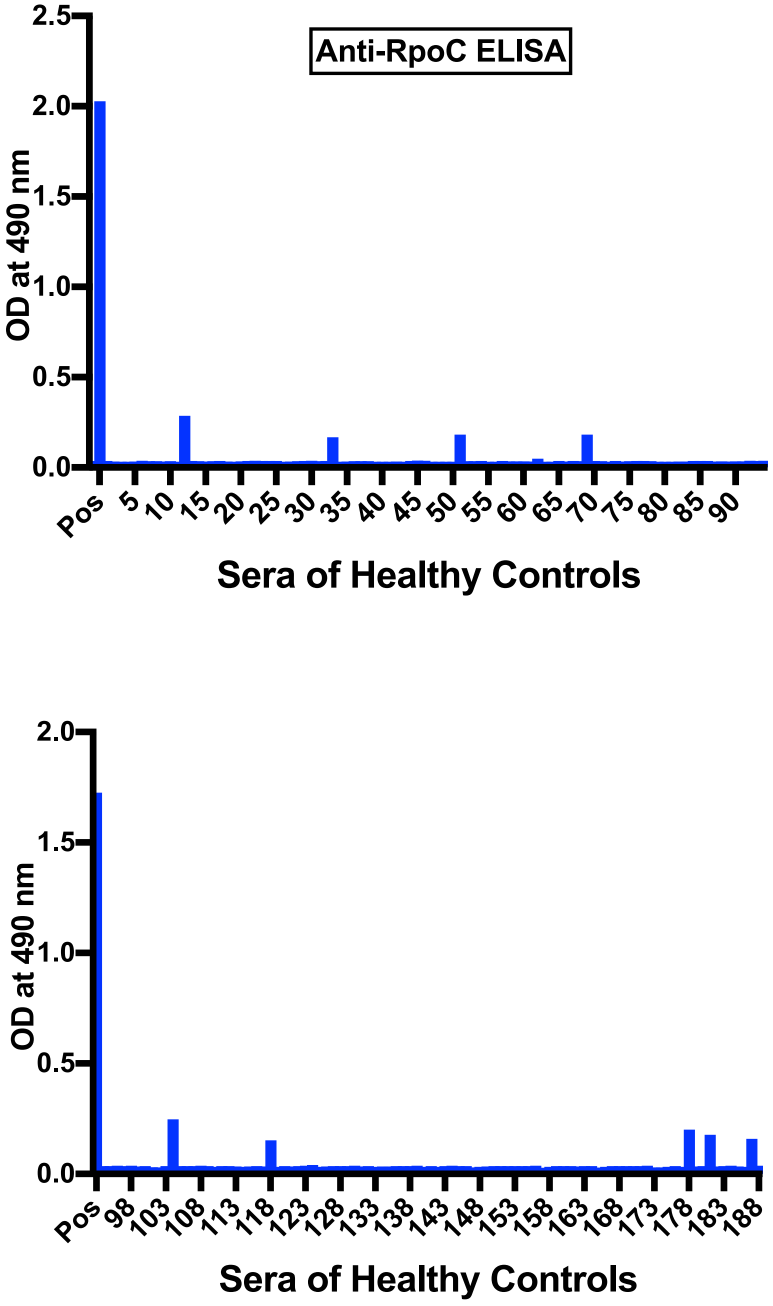

Supplementary Figure 8: ***M. catarrhalis*** **RpoC antibodies in serum.** Sera of healthy controls were tested for antibodies against RpoC of *M. catarrhalis*. Sera were diluted 1:100. The columns represent the measured OD. Independent experiments were repeated three times. One sero-positive NLPHL patient served as positive control.

A

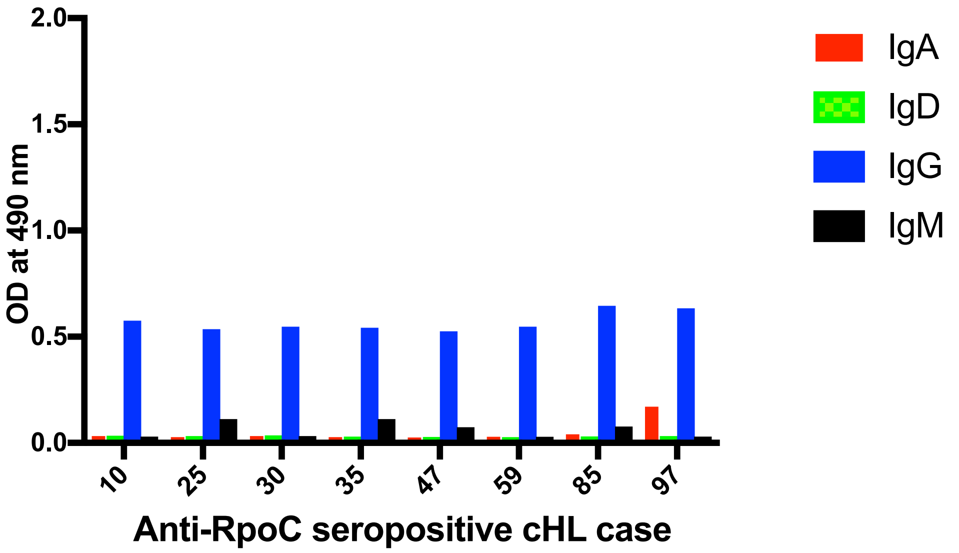

B

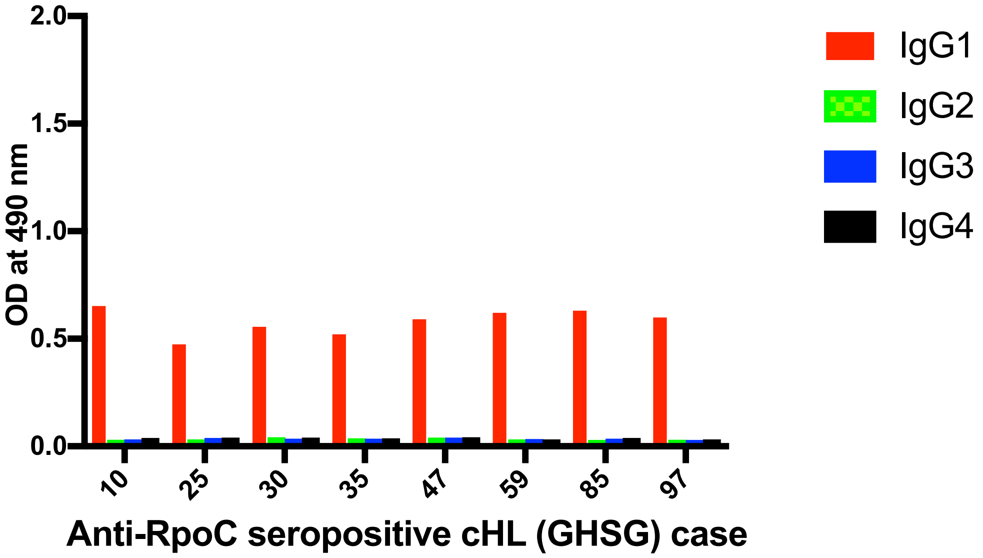

Supplementary Figures 9: Ig classes and IgG subclasses of *M. catarrhalis* RpoC antibodies in sera of NLPHL and cHL. ELISA for Ig class (A) and IgG subclass (B) of *M. catarrhalis* RpoC serum antibodies in seropositive patients with cHL. Sera were diluted 1:100. The columns represent the measured OD.

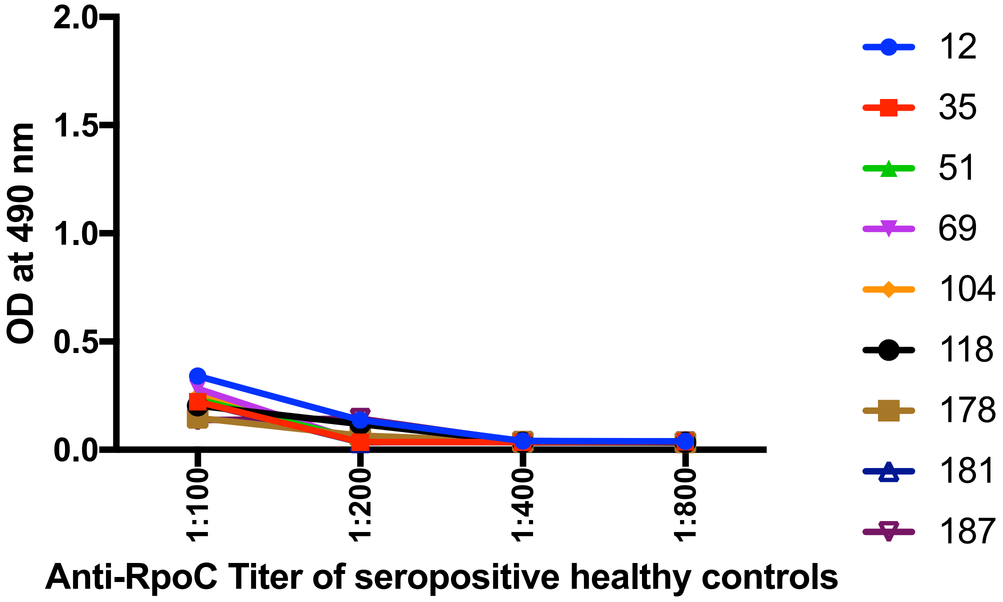

Supplementary Figure 10: Titers of *M. catarrhalis* RpoC antibodies in sera of seropositive healthy controls: The curves represent OD at 490 nm of different serum dilutions. Seropositive healthy controls have titers of up to 1:200.

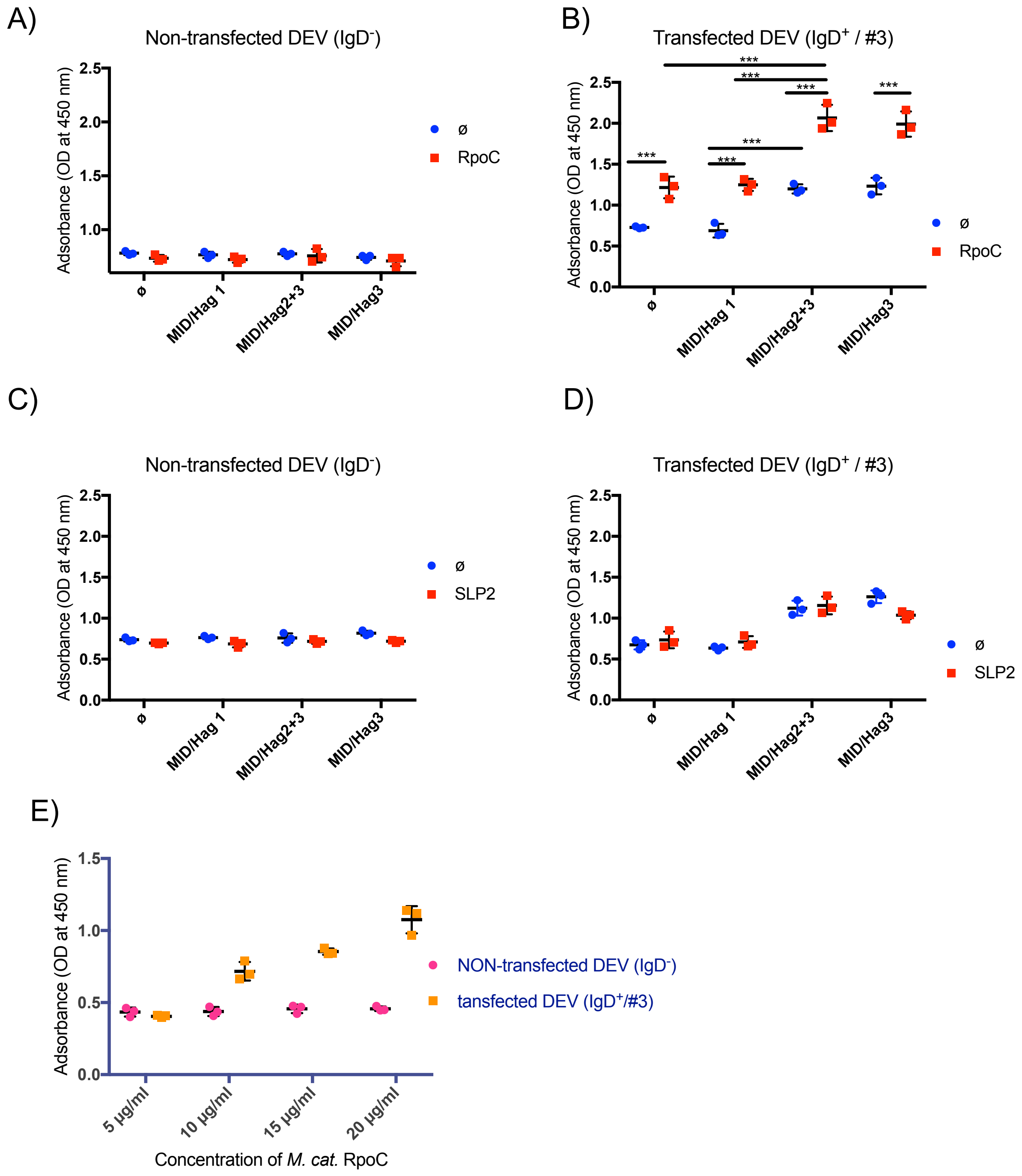

Supplementary Figure 11: Co-Stimulation of IgD-positive LP cells with different fragments of *Moraxella catarrhalis* MID/hag and *Moraxella catarrhalis* RpoC. A and B: Tetrazolium proliferation assays with a non-transfected DEV cell line (IgD$^-$) after addition of recombinant *Moraxella catarrhalis* RpoC (A) or recombinant human SLP2 (B) at 10 µg/ml and recombinant fragments of *Moraxella catarrhalis* MID/hag: MID/hag 1 (AA1-761), MID/hag 2 (AA920-1368) and MID/hag 3 (AA920-2090) at 10 µg/ml. After three days adsorbance of colored formazan as a marker for cell proliferation was detected at 450 nm. Neither human SLP2 nor *Moraxella catarrhalis* RpoC and MID/hag resulted in a growth stimulus. C and D:

The same proliferation assays performed with a DEV cell line transfected to express a *Moraxella catarrhalis* RpoC-reactive IgD^+ BCR. RpoC as well as MID/Hag fragments 2 and/or 3 induced a growth stimulus. MID/hag fragments 2 and 3 contain the IgD binding region (AA920-1200). Simultaneous incubation with RpoC and MID/hag fragments 2 and/or 3 showed an additive effect on proliferation of LP cells. Values are mean ± SD. For analysis of normal distribution the Kolmogorov-Smirnov test was applied, and statistical significance was tested by one-way analysis of variance (ANOVA) with Bonferroni correction for multiple testing, ** $p \leq 0.01$, *** $p \leq 0.001$, **** $p \leq 0.0001$. E: stimulation of growth by addition of RpoC to DEV cell line transfected to express a *Moraxella catarrhalis* RpoC-reactive IgD^+ BCR is dose dependant. Values are mean ± SD.

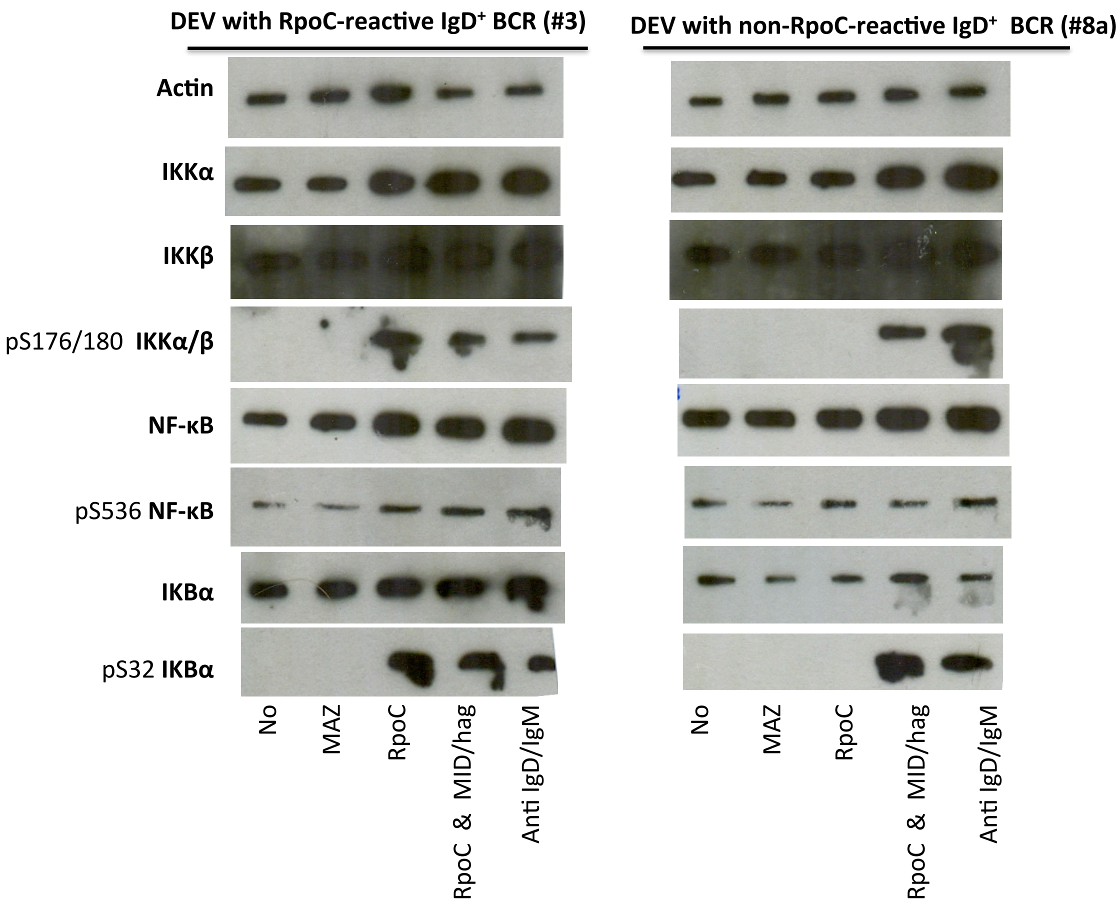

Supplementary Figure 12: Activation of the NF-κB signaling pathway. Representative Western blot analysis of components of the NF-κB signaling pathway shows activation by RpoC and even stronger activation by coincubation with RpoC and MID/hag in DEV cells transfected to express a RpoC-reactive IgD^+ BCR. NF-κB sampler kit CST (#9936). MAZ served as negative control. Data are representative of at 2 independent experiments

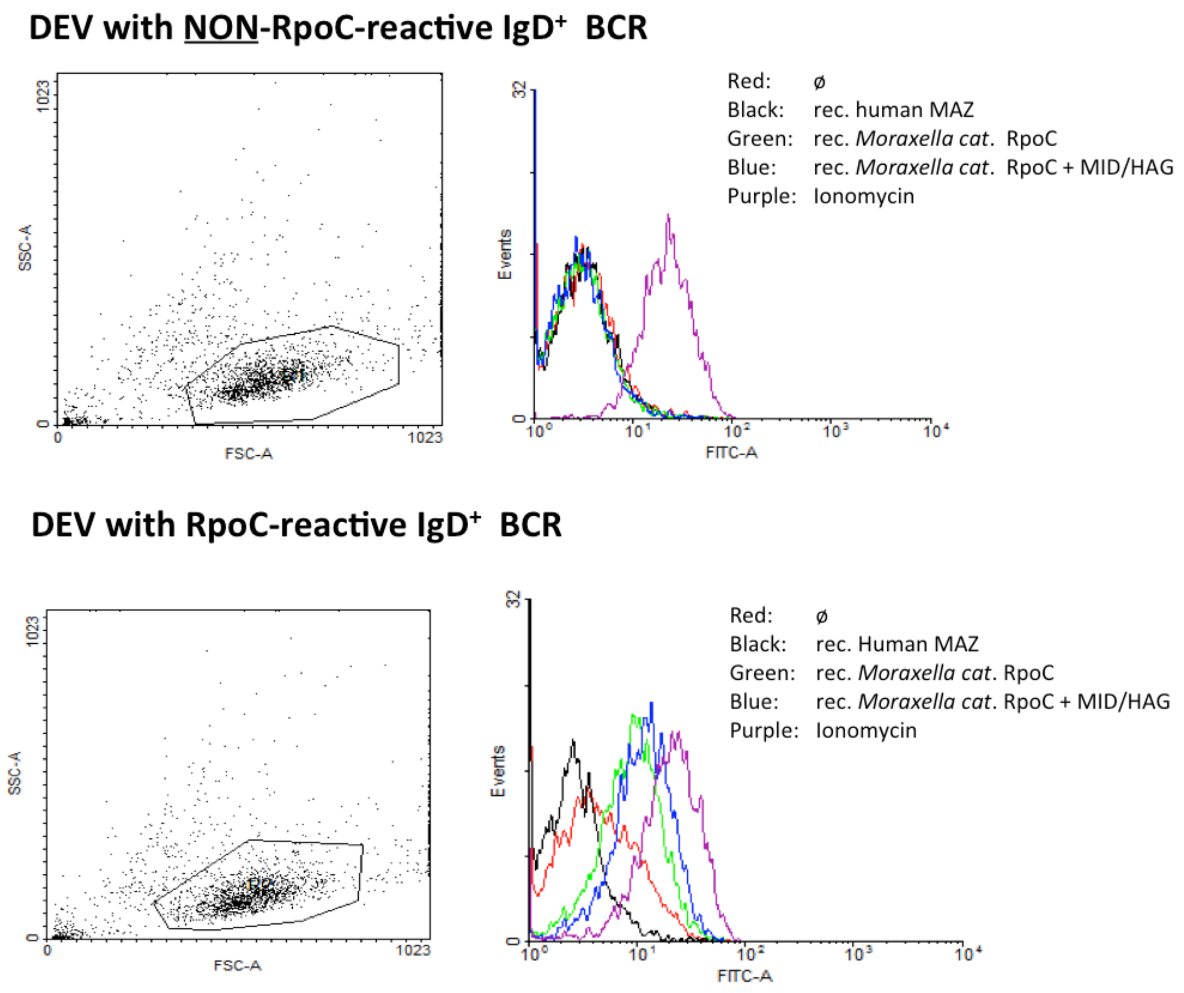

Supplementary Figure 13: Cytoplasmatic calcium levels in response to RpoC and RpoC and MID/Hag. Flow cytometric analysis of cytoplasmic calcium levels using Fluo-4d dye. On top, a DEV cell line transfected to express a RpoC-nonreactive IgD$^+$ BCR and on the bottom the DEV cell line transfected to express a RpoC-reactive IgD$^+$ BCR. The addition of *M. catarrhalis* RpoC and MID/hag led to an increase of intracellular calcium in the DEV cells expressing RpoC-reactive and IgD$^+$ BCR, indicating activation. Ionomycin served as positive control. Data are representative of 3 independent experiments.

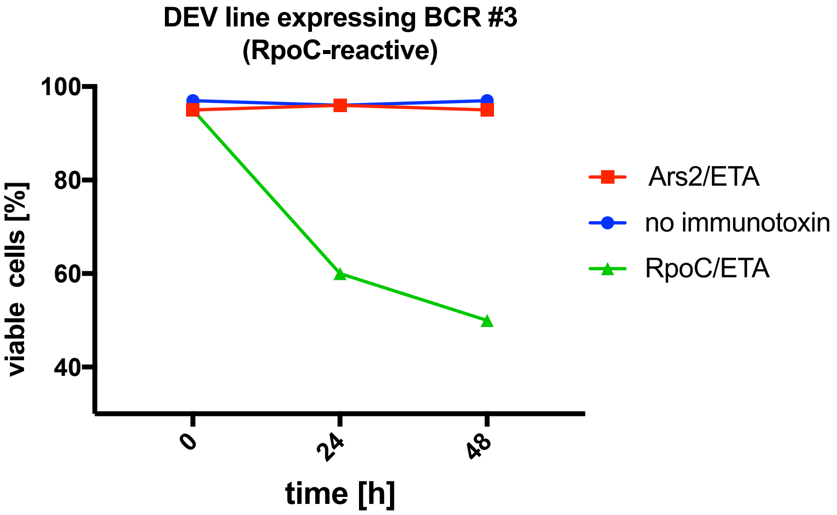

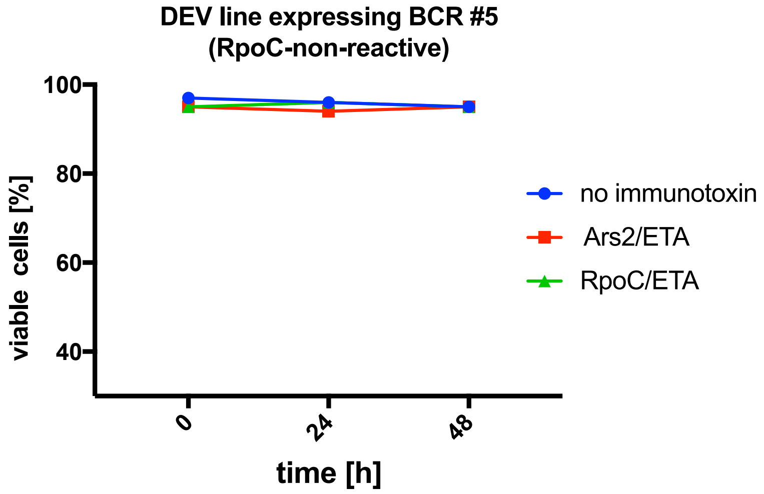

Supplementary Figure 14: Cytotoxic effects by ETA' toxin-conjugated RpoC dependent on expression of RpoC-reactive BCRs on LP cells. Cell viabilities of transfected DEV cells either expressing an RpoC-reactive BCR of patient #3 (above) or expressing a RpoC non-reactive, autoreactive BCR of #5 (below) after incubation with incubation with 5 µg/mL RpoC/ETA, Ars2/ETA as control toxin, or no immunotoxin were determined by trypan blue staining after 24 h and 48 h. Data are representative of 2 independent experiments.

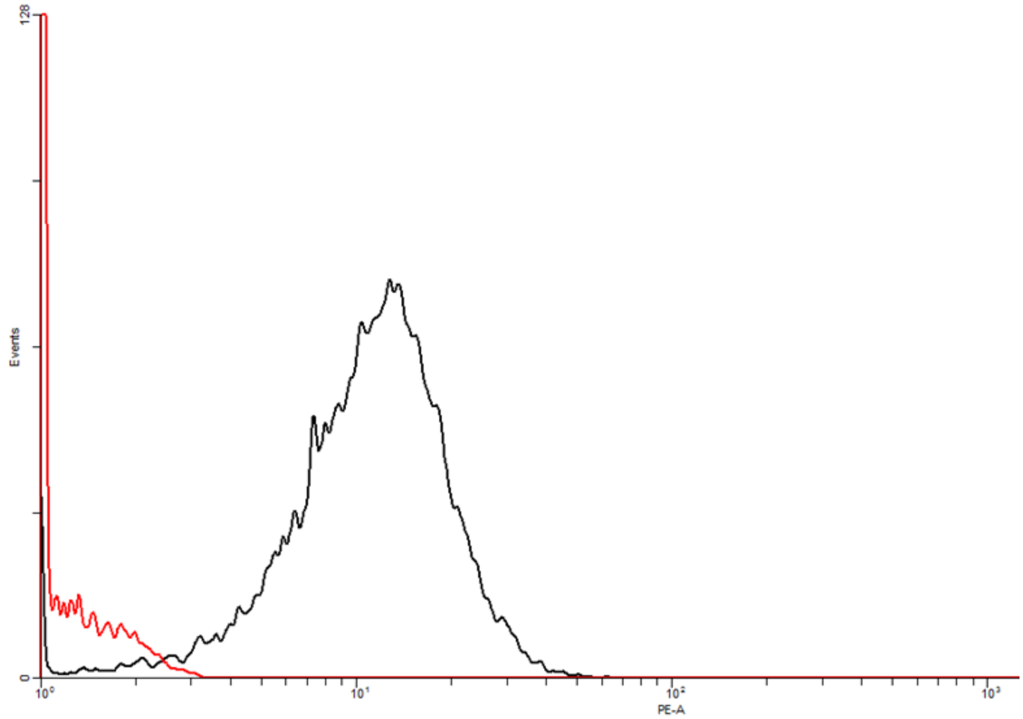

Supplementary Figure 15: Expression of recombinant patient derived BCR on DEV cell line. Flow cytometry for surface expression of recombinant His6 tagged BCR on transfected DEV cell line (black) or non-transfected DEV cell line (red) by PE anti His6-antibody. Data is representative of 3 independent experiments.

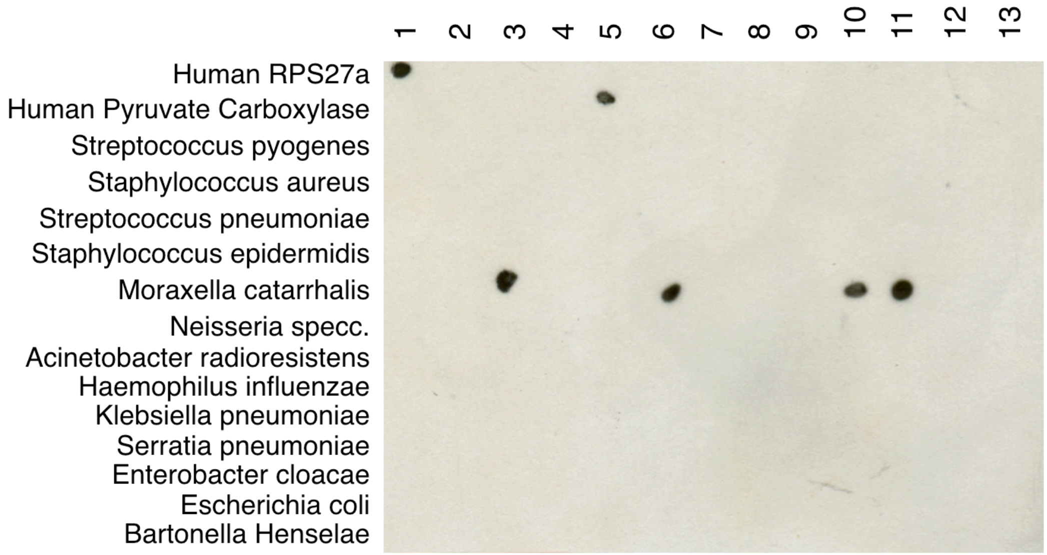
1 2 3 4 5 6 7 8 9 10 11 12 13
Human RPS27a
Human Pyruvate Carboxylase
Streptococcus pyogenes
Staphylococcus aureus
Streptococcus pneumoniae
Staphylococcus epidermidis
Moraxella catarrhalis
Neisseria specc.
Acinetobacter radioresistens
Haemophilus influenzae
Klebsiella pneumoniae
Serratia pneumoniae
Enterobacter cloacae
Escherichia coli
Bartonella Henselae

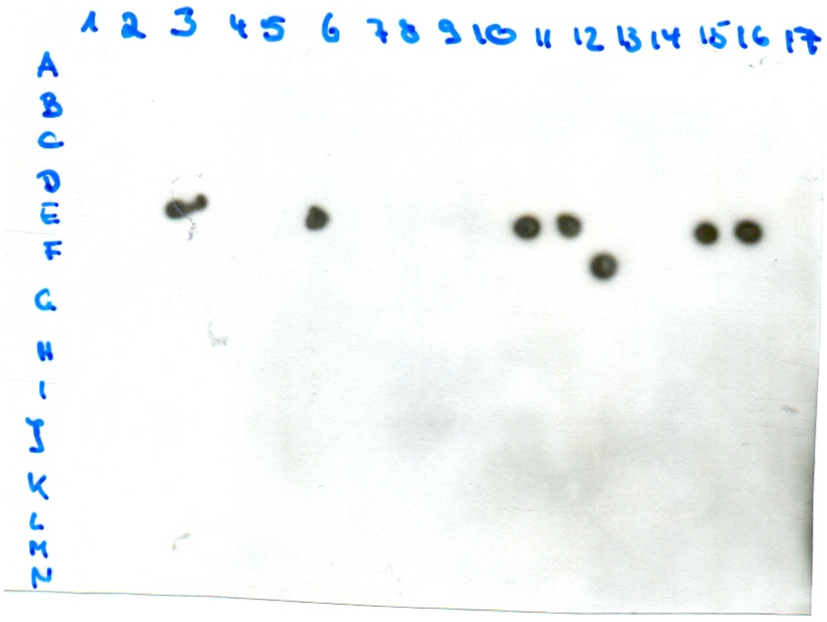
1 2 3 4 5 6 7 8 9 10 11 12 13 14 15 16 17
A
B
C
D
E
F
G
H
I
J
K
L
M
N

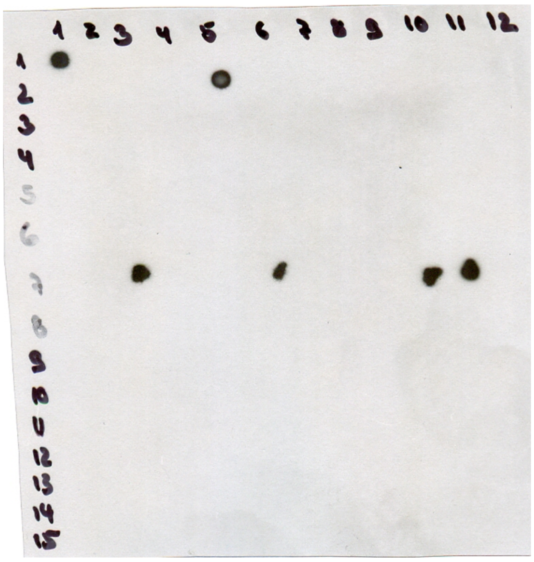

Supplementary Figure 16: Representative dot blots. Different representative examples of dot blots of lysates bacterial strains (Y-axis) and rec. Fabs of NLPHL (x-axis). The written numbers of NLPHL Fabs on the dot blots do not always correlate with case numbers in the manuscript! In the beginning Fabs of fewer cases were tested. In one of the examples rec. Fabs of 2 cases DLBCL were included to 15 NLPHL cases.

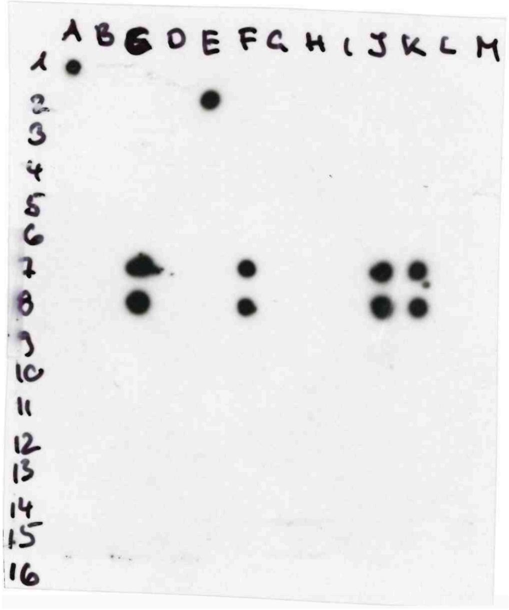

Supplementary Figure 17: Representative dot blot with two sources of lysate of *M. catarrhalis.* Another representative examples of dot blots of lysates bacterial strains (Y-axis) and rec. Fabs of NLPHL (x-axis). 6 and 7 represent lysates of *Moraxella catarrhalis* patient isolate and of *Moraxella catarrhalis* RO 108 ATCC 43617.

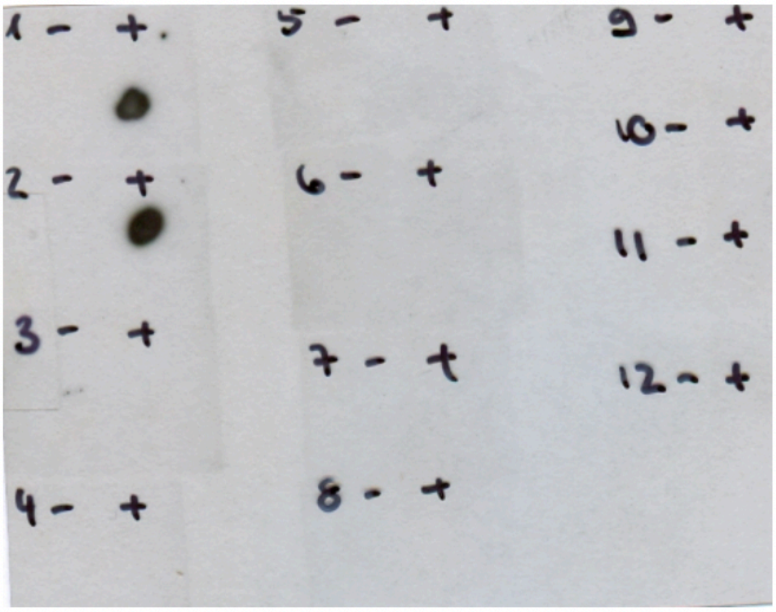

Supplementary Figure 18: Representative dot blot with PCNSL Fabs. As a control for specifity rec. Fabs of PCNSL were screened against M. catarrhalis RpoC. 1 and 2 were IgD^+ NLPHL cases (#3 and #6) as positive controls. PCNSL was chosen as it is regularly IgD^+IgM^+.

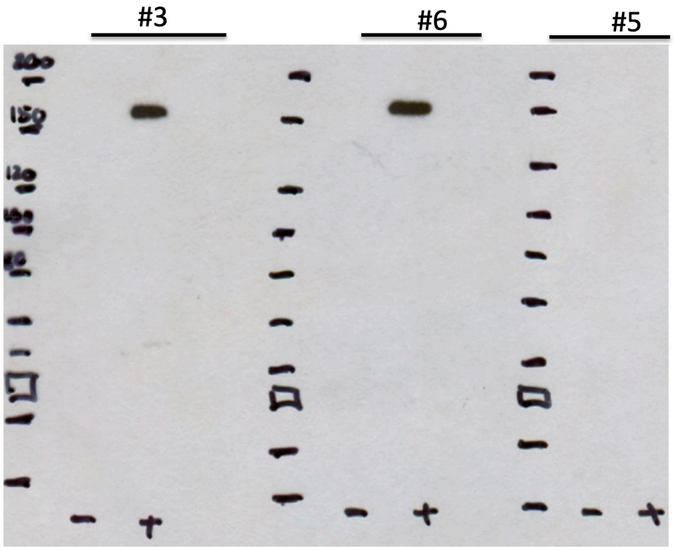

Supplementary Figure 19: Representative Western blots of *M. catarrhalis* and *K. pneumoniae* lysate using rec. Fabs of NLPHL as primary antibodies showing a band of 150 kDa as target.

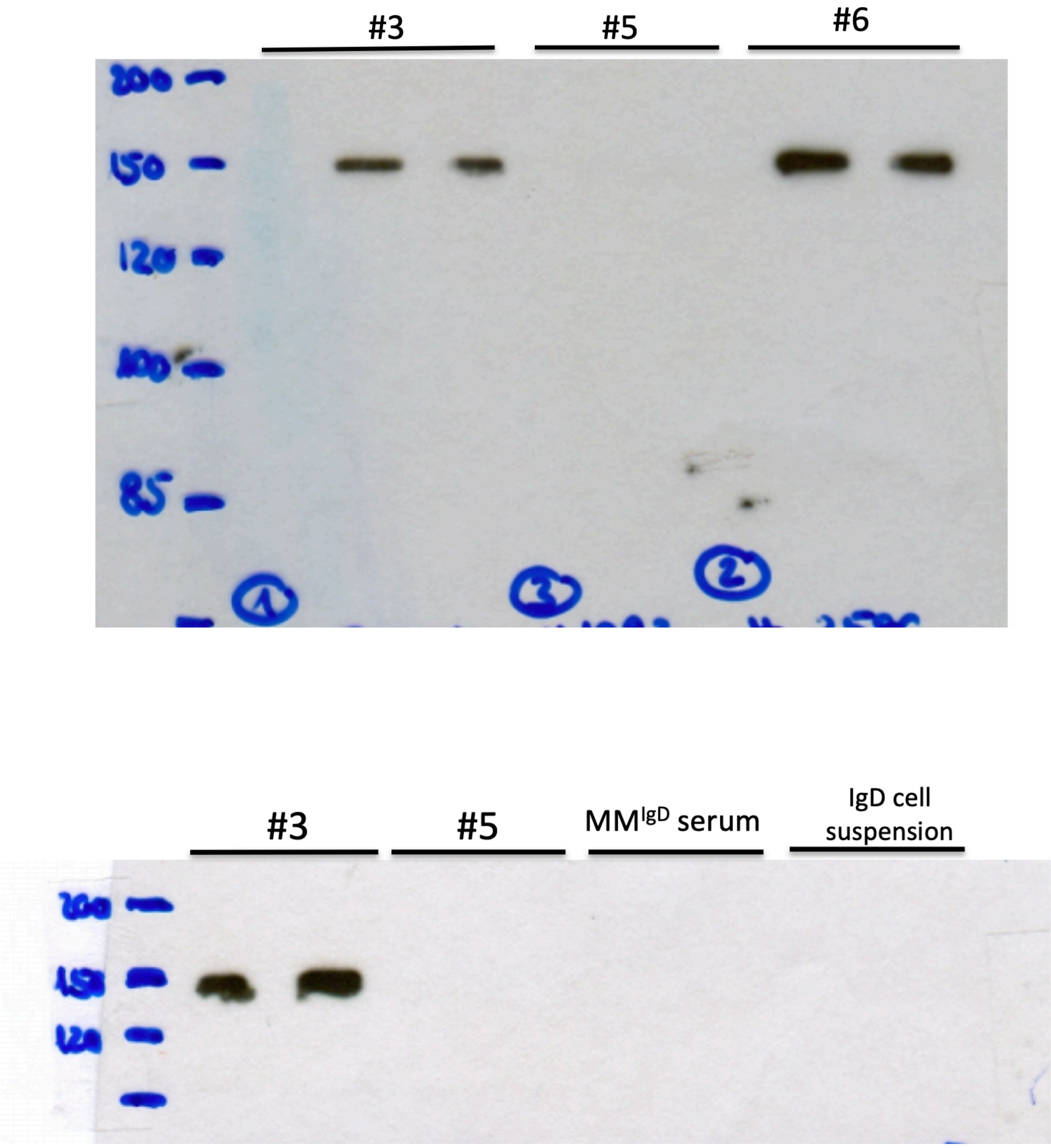

Supplementary Figure 19: Representative Western blot of lysates of *M. catarrhalis* using NLPHL derived Fabs as primary antibodies. 10% SDS gel, reducing conditions. Heat inactivated lysates of *Moraxella catarrhalis patient* lysates from Homburg and ATCC RO 108 ATCC 43617were compared. Fabs of #5 both *Moraxella catarrhalis* lysates. Fabs #5 did not bind any *Moraxella catarrhalis lysate, as they are reactive against* human pyruvate carboxylase. Neither IgD myeloma cell line supernatant nor IgD myeloma serum bound to the 150 kDa target of *Moraxella catarrhalis lysate*.

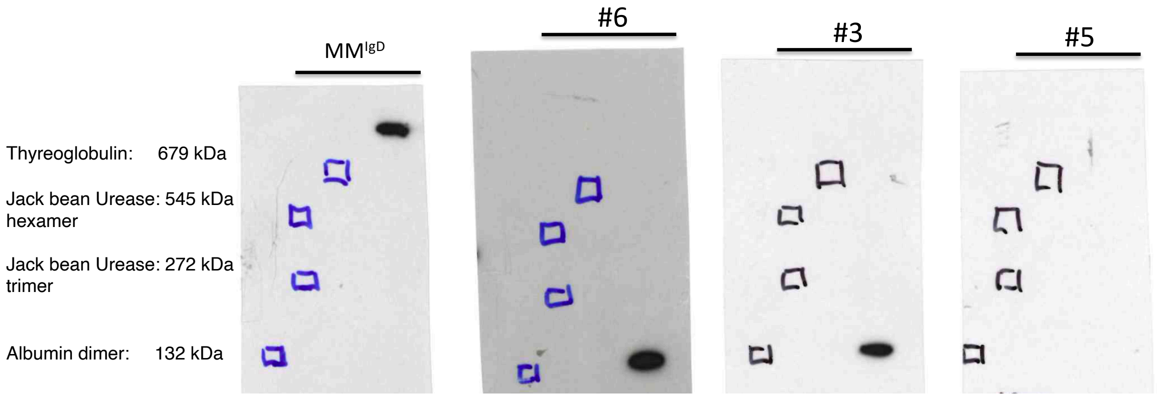

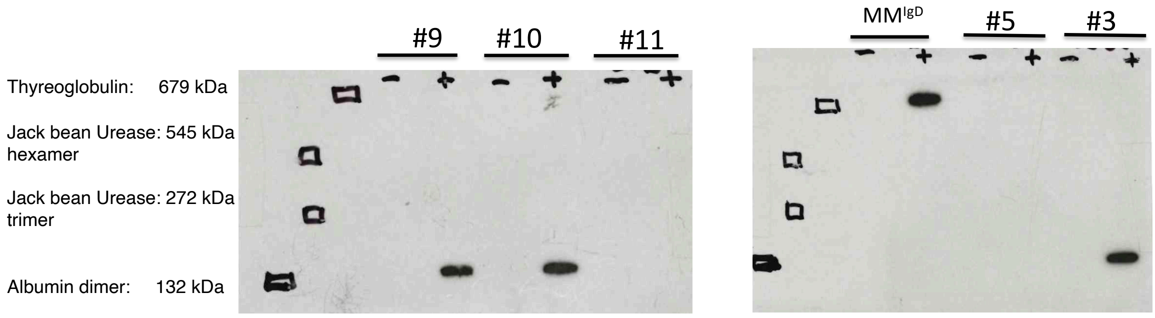

Supplementary Figure 20: Representative Western blot of lysates of *M. catarrhalis* showing a specific target antigen. Representative Western blots of lysates of *K. pneumoniae* and *M. catarrhalis* using rec. Fabs of NLPHL as primary antibodies. Sera of IgD multiple myeloma binds to as predicted to MID/hag tetramer but not the target of the NLPHL Fabs.

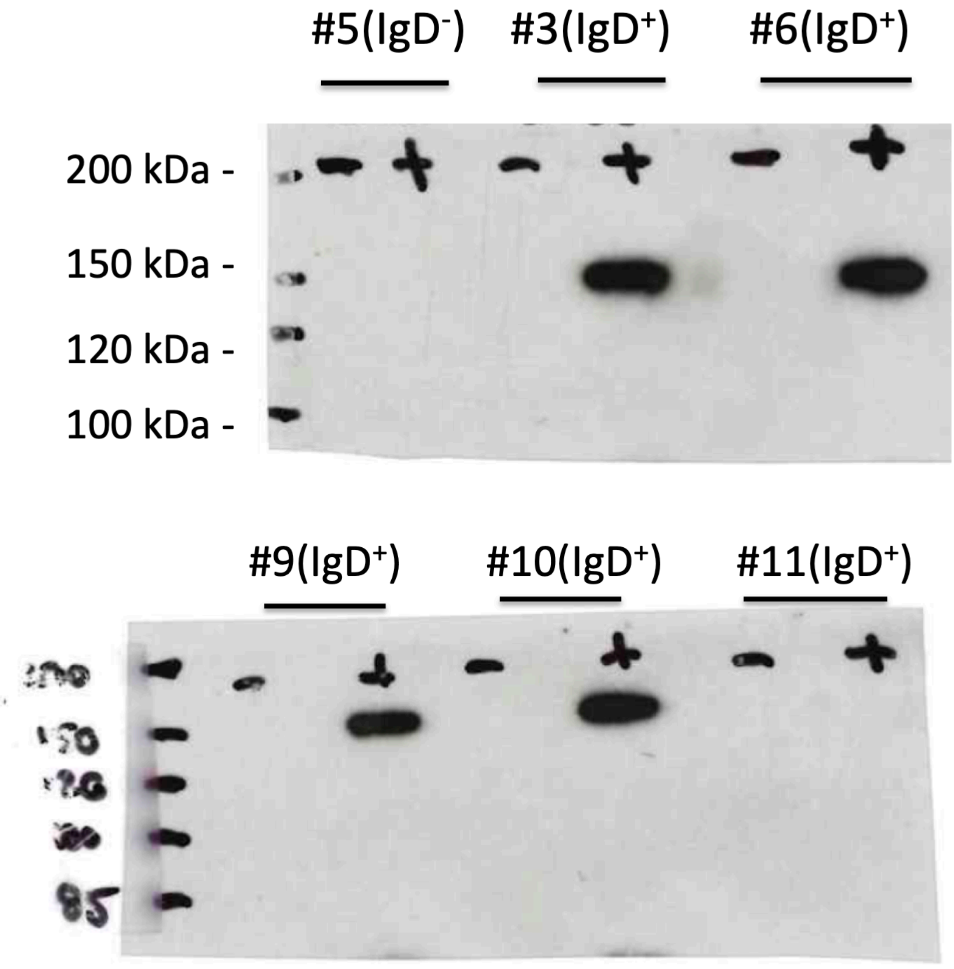

Supplementary Figure 21: Representative Western blot of lysates of *M. catarrhalis* using NLPHL derived Fabs as primary antibodies. Lysates of *K. pneumoniae* and *M. catarrhalis* using rec. Fabs of NLPHL as primary antibodies.

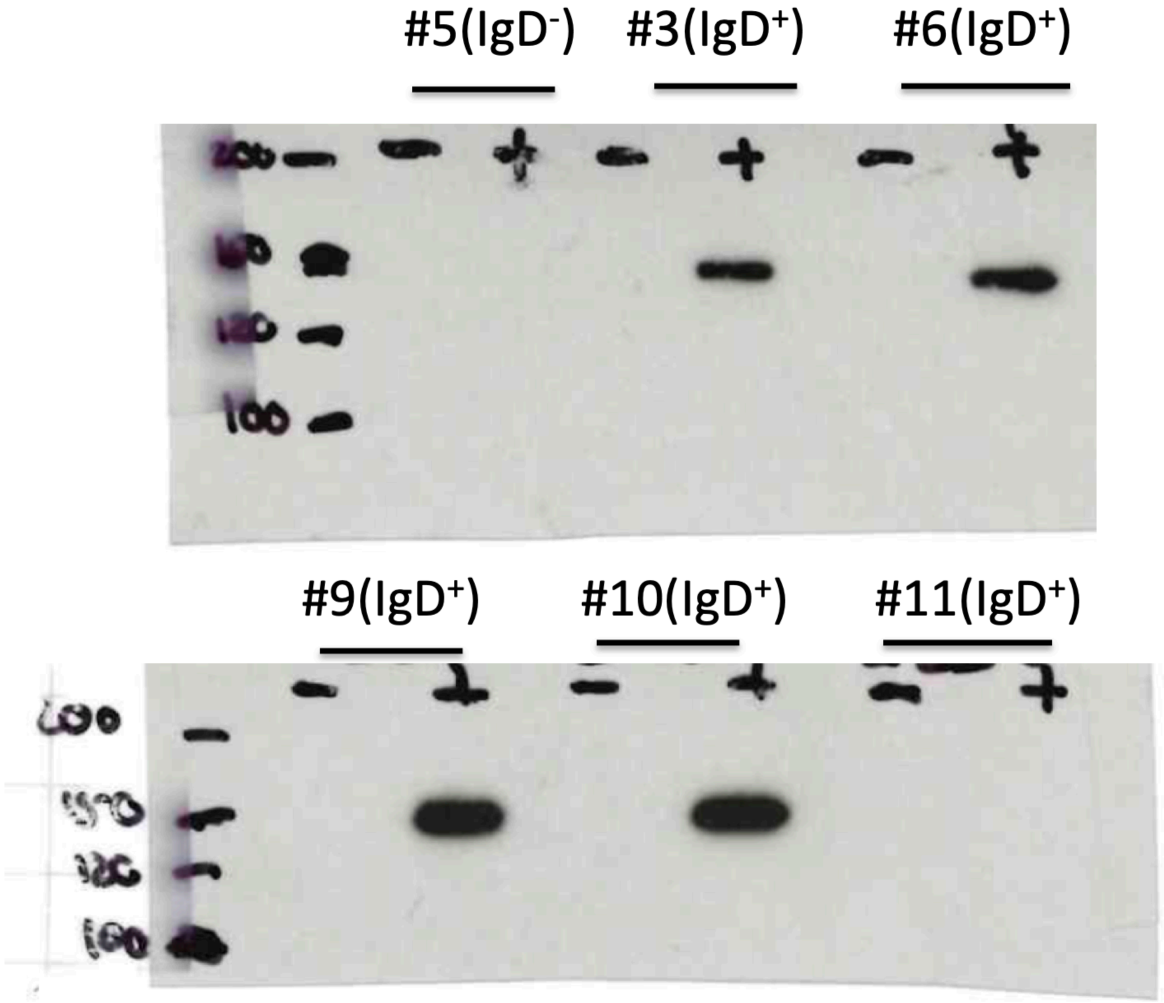

Supplementary Figure 22: Representative Western blot of rec RpoC of *M. catarrhalis* using NLPHL derived Fabs as primary antibodies. Representative Western blots of rec. RpoC of *M. catarrhalis* using rec. Fabs of NLPHL as primary antibodies.
